# Chest and Abdominal Wall Mobility and Cardiac Size in Older Adults With Heart Failure and Restrictive Spirometric Patterns: A Preliminary Cross-Sectional Study

**DOI:** 10.7759/cureus.105871

**Published:** 2026-03-26

**Authors:** Hideo Kaneko, Daiki Arahira, Akari Suzuki, Motonori Imazuru

**Affiliations:** 1 Department of Physical Therapy, Graduate School of Health and Welfare Sciences, International University of Health and Welfare, Okawa, JPN; 2 Department of Rehabilitation, Takagi Hospital, Okawa, JPN; 3 Department of Physical Therapy, School of Health Sciences at Fukuoka, International University of Health and Welfare, Okawa, JPN

**Keywords:** cardiac size, chest wall mobility, heart failure, older, restrictive spirometric pattern

## Abstract

Background: Restrictive spirometric patterns are common in older patients with heart failure (HF), and cardiac enlargement is considered a contributing factor. However, in older adults, reduced chest wall mobility primarily contributes to restrictive spirometric patterns. This preliminary study investigated the relationship of restrictive spirometric patterns with cardiac size and chest and abdominal wall mobility in hospitalized older patients with stable HF.

Methods: We conducted a preliminary cross-sectional study of older patients with stable HF who were hospitalized for an acute exacerbation of HF at Takagi Hospital in Japan, from December 2020 to November 2021. Restrictive spirometric patterns, cardiac size (assessed via cardiothoracic ratio), chest and abdominal wall mobility (evaluated by chest and total scale values of the breathing movement scale), physical performance (measured by the Short Physical Performance Battery), and cognitive function (evaluated by the Revised Hasegawa’s Dementia Scale) were examined in 65 hospitalized older patients with HF. The Mann-Whitney U test and Chi-squared test were used to compare patients with and without restrictive spirometric patterns. In addition, univariable and multivariable logistic regression analyses were performed to assess whether cardiothoracic ratio, chest, or total scale values were associated with restrictive spirometric patterns.

Results: Twenty-one patients were excluded (patients with a neurological disease, dementia, or airflow limitation). Of the 44 patients (23 females), 20 had restrictive spirometric patterns. Patients with restrictive spirometric patterns had lower values on the chest and total scales, the Short Physical Performance Battery, and the Revised Hasegawa’s Dementia Scale than patients without restrictive spirometric patterns. There were no significant differences between the groups in basic characteristics. Multivariable logistic regression analysis indicated that both the chest and total scale values were significantly associated factors of restrictive spirometric patterns (chest scale: odds ratio = 0.59; 95% confidence interval = 0.39−0.82, total scale: odds ratio = 0.64; 95% confidence interval = 0.45−0.83).

Conclusion: Our findings suggest that restrictive spirometric patterns may be associated with reduced chest and abdominal wall mobility in hospitalized older patients with stable HF.

## Introduction

Among older adults, heart failure (HF) is the most common cause of hospitalization [1] and has several risk factors, such as advanced age, kidney disease, diabetes, and chronic obstructive pulmonary disease [2], which increase the mortality risk in patients with HF [2-5]. Recent studies have indicated that restrictive ventilatory impairments are common in patients with HF and are associated with mortality [6,7]; moreover, restrictive spirometric patterns (RSPs) are a risk factor for incident HF in older adults [8].

An RSP is usually assessed using spirometric parameters such as forced vital capacity (FVC) and forced expiratory volume in 1 second (FEV_1_). Reduced FVC and FEV_1_ in patients with HF could be attributed to HF-induced pulmonary congestion and cardiac enlargement [9,10]. Cardiac enlargement and pulmonary congestion may restrict inspiration by reducing thoracic and pulmonary compliance. Furthermore, the prevalence of RSPs in older adults is >10% [11,12]. Reduced chest wall mobility significantly contributes to RSPs in older adults [13]. Therefore, reduced chest wall mobility may also contribute to RSPs in older adults with HF. However, chest and abdominal wall mobility in patients with HF remains unclear, and the relationship of RSPs with chest and abdominal wall mobility in older patients with HF remains unclear.

Therefore, we conducted a preliminary study to examine whether chest and abdominal wall mobility or cardiac size might be related to RSPs in older patients with HF. We hypothesized that reduced chest and abdominal wall mobility would be more closely associated with RSPs than cardiac size.

This article was previously presented as a meeting abstract at the 38th International Meeting of Physical Therapy Science in Korea on September 5-7, 2025 [14].

## Materials and methods

Study design and participants

We conducted a preliminary cross-sectional study of older patients with stable HF who were hospitalized for an acute exacerbation of HF at Takagi Hospital in Japan, from December 2020 to November 2021. The inclusion criteria were patients with stable cardiac function without obvious HF symptoms, including dyspnea and edema. The exclusion criteria were as follows: patients who had undergone thoracic or abdominal surgery within the previous 2 months, as well as patients with neurological disease, dementia, or airflow limitation. This study was approved by the Ethics Committee of International University of Health and Welfare (approval number: 20-Ifh-063) on November 24, 2020, and written informed consent was obtained from all participants.

Demographic and clinical data

Clinical data were obtained from medical records, including age, sex, body mass index, etiology of HF, echocardiographic findings, cardiac size, and Revised Hasegawa’s Dementia Scale (HDS-R) scores. These data were collected within one week before discharge. The Short Physical Performance Battery (SPPB), spirometry, and chest and abdominal wall mobility were assessed by a single examiner within three days before discharge.

Echocardiogram

Left ventricular ejection fraction on two-dimensional echocardiogram was defined as follows: HF with reduced ejection fraction for <40%, HF with mid-range ejection fraction for 40%-49%, and HF with preserved ejection fraction for ≥50%.

Cardiac Size

Cardiac size was assessed using the cardiothoracic ratio, which was defined as the ratio of the transverse diameter of the heart to the transverse diameter of the thoracic cavity on chest radiography. All X-ray images were taken in a standing posture under inspiratory conditions.

Short Physical Performance Battery

The SPPB has three components: the balance test, the five-time chair stand test, and the 4-meter walk test [15]. For the balance test, the patient was required to maintain three positions (feet placed side-by-side, semi-tandem, and tandem) for 10 seconds per position. The 4-meter walk test was performed twice at normal walking speed, with the shorter time being recorded. In the five-time chair stand test, the patient was instructed to promptly complete five chair stands with the arms folded across the chest. Each test was scored from 0 (worst) to 4 (best), with the total score ranging from 0 to 12.

Revised Hasegawa’s Dementia Scale

The HDS-R is a cognitive screening tool consisting of nine questions, for a maximum score of 30 points. Dementia was defined as a score of <21 on the HDS-R according to a previous study [16].

Spirometry

FVC, FEV_1_, and FEV_1_/FVC were measured using a spirometer (Spirodoc, Medical International Research, Roma, Italy) based on the American Thoracic Society and European Respiratory Society guidelines [17]. The lower limit of normal (LLN) at the fifth percentile of distribution for FVC, FEV_1_, and FEV_1_/FVC was based on equations derived by the Japanese Respiratory Society using the Lambda-Mu-Sigma method [18]. FVC was expressed as a percentage of predicted FVC and Z-score. RSPs were indicated by FVC < LLN and FEV_1_/FVC ≥ LLN. A Z-score of -1.645 indicates the LLN according to the American Thoracic Society and European Respiratory Society guidelines recommendation [19].

Chest and abdominal wall mobility

Chest and abdominal wall mobility were assessed using the breathing movement scale for deep breathing. The breathing movement scale assesses chest and abdominal wall movements during deep breathing on a 0-8 scale, with acceptable reliability and validity [20]. A breathing movement scale value <4 indicates reduced mobility below the lower limit of deep breathing movement in healthy adults. According to a previous study [13], deep breathing movements were observed at five measurement points using the breathing movement measuring device (BMMD, Fukuoka, Japan): the upper and lower chest on each side and the abdomen. In cases of no obvious asymmetry in chest breathing movements between the right and left sides, deep breathing movements were measured at three measurement points: the right upper chest (third rib), right lower chest (eighth rib), and upper abdomen (above the umbilicus), using a breathing movement-measuring device. In cases where a discrepancy greater than one scale value was observed in the left-right asymmetry of chest breathing movements, the mean of the scale values from the left and right sides of the chest was employed. Measurements were performed twice, with the maximum values being recorded. The chest scale value (0-16) was obtained as the sum of the upper and lower chest scale values. Subsequently, the chest and abdominal scale values were summed to obtain the total scale value (0-24). In older adults, cutoff values of 7 and 12 (11 for females) for the chest and total scales, respectively, have been suggested for screening RSPs [13]. These cutoffs were used to define reduced chest and abdominal wall mobility.

Sample size

The sample size was calculated based on previous studies that reported large effect sizes for differences between RSPs and chest wall mobility [13] as well as relationships between FVC and cardiac enlargement [10]. We considered Cohen’s d = 0.8, α = 0.05, power = 0.8, and allocation ratio = 0.8 for differences between two independent means. The estimated sample size was 42, and with a 40% attrition rate (due to dementia or airflow limitation), the sample size was set at 59.

Statistical analysis

All statistical analyses were performed using IBM SPSS Statistics for Windows version 27 (IBM, Armonk, NY, USA). Continuous variables were expressed as means and standard deviations, ordinal variables were expressed as medians and interquartile ranges, and categorical variables were expressed as numbers. Participants were divided into those with and without RSPs (RSP and non-RSP groups, respectively). Between-group comparisons were performed using the Chi-squared test for categorical variables, unpaired t-test for continuous variables, and Mann-Whitney U test for ordinal variables and continuous variables with non-normal distribution based on the Shapiro-Wilk test. Univariable and multivariable logistic multiple regression analyses were used to determine whether the cardiothoracic ratio, chest scale, or total scale was associated with RSPs. All tests were performed using a two-tailed test, with statistical significance set at p < 0.05.

## Results

We included 65 hospitalized patients with HF; among them, we excluded 21 patients (three with a neurological disease, ten with dementia, and eight with airflow limitation). Of the subjects, 20 and 24 were divided into RSP and non-RSP groups, respectively. The RSP group had significantly lower FVC and FEV_1 _(both % predicted and Z-score), chest and total scale values, SPPB scores, and HDS-R scores than the non-RSP group (Table 1). Eleven and 16 patients in the RSP group had reduced chest and total scale values, respectively, which were significantly higher than those of the non-RSP group (1 and 4 for the chest and total scale values, respectively). There were no significant between-group differences in age, sex, body mass index, HF etiology, cardiac function, or cardiothoracic ratio.

**Table 1:** Cardiopulmonary, physical, and cognitive functions in hospitalized older patients with stable heart failure a: Sum of scale scores of upper and lower chest on the breathing movement scale. b: Sum of scale scores of upper chest, lower chest, and abdomen on the breathing movement scale. Between-group comparisons were performed using the Chi-squared test for categorical variables, unpaired t-test for continuous variables, and Mann–Whitney U test for ordinal variables and continuous variables with non-normal distribution based on the Shapiro–Wilk test. RSP, restrictive spirometric pattern; LVEF, left ventricular ejection fraction; HFrEF, heart failure with reduced ejection fraction; HFmrEF, heart failure with mid-range ejection fraction; HFpEF, heart failure with preserved ejection fraction; FVC, forced vital capacity; FEV1, forced expiratory volume in 1 second; SPPB, Short Physical Performance Battery; HDS-R, Revised Hasegawa's Dementia Scale

Variables	Total (N = 44)	RSP group (n = 20)	Non-RSP group (n = 24)	p-value
Age (years), mean ± SD	80 ± 7	82 ± 8	79 ± 6	0.154
Male/Female, n	21/23	11/9	10/14	0.378
Height (m), mean ± SD	1.55 ± 0.08	1.55 ± 0.09	1.54 ± 0.09	0.863
Weight (kg), mean ± SD	51.5 ± 9.0	50.2 ± 8.7	52.6 ± 9.2	0.386
Body mass index (kg/m^2^), mean ± SD	21.6 ± 3.5	21.0 ± 3.4	22.1 ± 3.6	0.961
Heart failure etiology				0.954
Ischemic heart disease, n	6	3	3	
Valvular heart disease, n	25	11	14	
Cardiomyopathy, n	3	1	2	
Others, n	10	5	5	
Cardiothoracic ratio (%), mean ± SD	0.54 ± 0.06	0.55 ± 0.06	0.54 ± 0.06	0.624
LVEF (%), mean ± SD	45.8 ± 17.0	44.0 ± 15.7	47.3 ± 18.2	0.529
HFrEF/HFmrEF/HFpEF, n	15/6/23	7/4/9	8/2/14	0.48
FVC % predicted, mean ± SD	78.2 ± 23.5	58.7 ± 14.3	94.4 ± 16.2	<0.001
FVC Z-score, median (IQR)	-1.55 (-2.76 to -0.39)	-2.92 (-3.39 to -2.19)	-0.40 (-1.08 to -0.11)	<0.001
FEV_1 _% predicted, mean ± SD	79.1 ± 44.0	63.0 ± 20.0	92.0 ± 24.0	<0.001
FEV_1_ Z-score, median (IQR)	-1.22 (-2.19 to -0.30)	-2.44 (-3.28 to -1.71)	-0.21 (-0.95 to 0.21)	<0.001
FEV_1_/FVC (%), mean ± SD	79.7 ± 6.4	80.2 ± 7.0	79.2 ± 6.0	0.637
FEV_1_/FVC Z-score, mean ± SD	0.46 ± 1.00	0.57 ± 1.09	0.37 ± 0.95	0.507
Breathing movement scale				
Chest scale^a^ (0–16), median (IQR)	8 (7–10)	6 (5–7)	9 (7–10)	<0.001
Reduced mobility (<7), n	12	11	1	<0.001
Total scale^b^ (0–24), median (IQR)	13 (10–15)	10 (7–11)	13 (11–15)	0.001
Reduced mobility (males <12, females <11), n	20	16	4	<0.001
SPPB (0–12), median (IQR)	10 (8–11)	9 (4–11)	11 (8–12)	0.033
HDS-R (0–30), mean ± SD	25.9 ± 3.1	24.8 ± 2.9	26.8 ± 2.9	0.023

In the univariable logistic regression analysis, the chest and total scale values were significant predictors of RSPs (Table 2). In the multivariable logistic regression analysis, both the chest and total scale values remained significant predictors of RSPs (chest scale: odds ratio = 0.59; 95% confidence interval = 0.39−0.82, total scale: odds ratio = 0.64; 95% confidence interval = 0.45−0.83).

**Table 2:** Associations of cardiothoracic ratio and breathing movement scale scores with restrictive spirometric patterns in hospitalized older patients with heart failure a: Sum of scale scores of upper and lower chest on the breathing movement scale. b: Sum of scale scores of upper chest, lower chest, and abdomen on the breathing movement scale. Model 1: Independent variables from the chest scale. Model 2: Independent variables from the total scale.

Variables	Univariable analysis		Multivariable analysis	
	Odds ratio (95% CI)	p-value	Odds ratio (95% CI)	p-value
Model 1				
Cardiothoracic ratio	1.03 (0.93–1.13)	0.941	1.00 (0.89–1.11)	0.941
Chest scale^a^	0.60 (0.40–0.82)	0.005	0.59 (0.39–0.82)	0.005
Model 2				
Cardiothoracic ratio			1.00 (0.89–1.13)	0.997
Total scale^b^	0.64 (0.45–0.83)	0.003	0.64 (0.45–0.83)	0.003

## Discussion

This preliminary cross-sectional study of older patients with stable HF supports our hypothesis that reduced chest and abdominal wall mobility would be associated with RSPs rather than cardiac size. In hospitalized older patients with stable HF, no significant between-group difference was observed in the cardiothoracic ratio. However, both the chest and total scale values were found to be low, and these were identified as significant factors associated with RSPs. These findings suggest that, among hospitalized older adults with stable HF, RSPs may be more closely related to reduced chest and abdominal wall mobility than cardiac enlargement.

Approximately half of our included patients (20/44) had RSPs. A recent study reported that RSPs are common in patients with HF and reduced ejection fraction, with more than half of them having RSPs [7]. Similarly, another study reported that 44.6% of patients with HF and preserved ejection fraction without airflow limitation had FEV_1_ (% predicted) <80% [6]. Since these studies included many older patients with HF, it was assumed that similar results would be obtained in this study.

Both the chest and total scale values were significantly associated with RSPs. The close relationship of RSPs with chest wall mobility in hospitalized older patients with stable HF is consistent with that observed in older adults [13]. However, there was a higher percentage of patients with HF and reduced chest and abdominal wall mobility (20/44) than in previously reported older adults (37/154). This suggests that reduced abdominal wall mobility is more common in older patients with HF than in healthy older adults. Given the close relationship between abdominal wall mobility and diaphragmatic movement [21], older patients with HF might have diaphragmatic dysfunction, as previously suggested [22].

There was no significant between-group difference in cardiothoracic ratio, which did not support our hypothesis. The cardiothoracic ratio (54.2%) and FVC (78.2%) in the present study were comparable to previously reported values (57% and 73%, respectively) [23]. Notably, our patients were older and had different HF types, while those in the previous study were younger (62 years) and had HF with reduced ejection fraction. In this study, no differences in HF type were observed between the RSP and non-RSP groups. Therefore, it is considered that there is little association between HF types and RSPs in older patients with HF. Additionally, since cardiothoracic ratio is affected by ventilation [24], it is likely that the cardiothoracic ratio in older adults is affected by reduced chest wall mobility. In other words, a decrease in the transverse diameter of the thoracic cavity caused by reduced chest wall mobility may increase the cardiothoracic ratio. This may contribute to the lack of a relationship between RSPs and cardiothoracic ratio.

The RSP group had lower SPPB and HDS-R scores than the non-RSP group. Poor physical performance in the SPPB is associated with an increased mortality risk in older patients with HF [25]. Specifically, patients with HF who have an SPPB score <7 are considered to have a poor prognosis [26]. In this study, there were eight patients with an SPPB score <7, with seven of them being in the RSP group. Furthermore, considering that physical performance is associated with cognitive function rather than RSPs [27,28], SPPB scores may be related to HDS-R scores. These findings suggest a potential link between poor physical performance, cognitive decline, and RSPs in older patients with stable HF. Further research is needed to investigate the relationship between physical function and cognitive decline with RSPs.

To the best of our knowledge, this is the first study to demonstrate the relationship of RSPs with chest and abdominal wall mobility and cardiac enlargement in patients with HF. However, this study has several limitations. First, this was a small-scale observational study; that is, we were unable to account for relevant confounders, and we cannot infer a causal relationship between RSPs and chest and abdominal wall mobility. In addition, our findings may not apply to a broader population of older patients with HF. Furthermore, although RSPs were defined based on FVC, the absence of total lung capacity measurements means that they only suggest the presence of restrictive ventilatory impairment and do not prove it [29]. Finally, since cardiothoracic ratio measurements using chest radiography cannot assess changes in anterior-posterior heart size, cardiac enlargement may have been underestimated [30]. Large-scale longitudinal studies using more accurate diagnostic assessments are warranted to identify the factors associated with reduced lung volume in older patients with stable HF.

## Conclusions

This preliminary study demonstrated that in hospitalized older patients with stable HF, chest and total scale values, but not cardiothoracic ratio, were significantly associated factors of RSPs. These findings suggest that RSPs may be associated with reduced chest and abdominal wall mobility in older patients with stable HF. Large-scale longitudinal studies are needed to identify the factors associated with reduced lung volume in older patients with stable HF.
